# Bibliometric profile of the global scientific research on autism spectrum disorders

**DOI:** 10.1186/s40064-016-3165-6

**Published:** 2016-09-02

**Authors:** Waleed M. Sweileh, Samah W. Al-Jabi, Ansam F. Sawalha, Sa’ed H. Zyoud

**Affiliations:** 1 Department of Physiology, Pharmacology and Toxicology, College of Medicine and Health Sciences, An-Najah National University, Nablus, Palestine; 2 Department of Clinical and Community Pharmacy, College of Medicine and Health Sciences, An-Najah National University, Nablus, Palestine

**Keywords:** Autism spectrum disorder, Bibliometric analysis, VOSviewer, Worldwide

## Abstract

**Background:**

Autism spectrum disorders (ASD) are a group conditions classified as neuro-developmental disorders. Research activity on ASD is important for all countries since such disorders have both social and health consequences. The objective of this study was to analyze research output on ASD during the period 2005–2014.

**Methods:**

All articles relevant to ASD plus all articles published in autism journals were retrieved using Scopus database. VOSviewer software was used to create density and network visualization maps. Bibliometric indicators were investigated by analyzing annual research output, languages, countries, institutions, journals, title terms, highly cited articles, and co-authorship relations.

**Results:**

A progressive annual growth of ASD research was observed from 2005 to 2014. During this period, a total of 18,490 articles were retrieved. The majority of these articles was published in *Journal of Autism and Developmental Disorders* with 48,416 citations and an average citation of 23.59 citations per article. The countries that published the largest number of articles were the United States (US) (*n* = 8594; 46.48 %), United Kingdom (*n* = 2430; 13.14 %) and Canada (*n* = 1077; 5.8 %). International collaborations produced 30.18 % of the articles published by top 10 productive countries. King’s College London (UK) ranked first in number of publications and total citations. The top 10 list of productive institutions was dominated by US academic and research institutions. More than half of the highly cited articles were in the field of molecular genetics. Articles with more than 50 citations were published mainly by authors from USA, UK and Canada.

**Conclusions:**

There is a worldwide growth of publications on ASD led by countries in Northern America and Europe. Retrieved articles were published in a wide range of journals. Molecular genetics of ASD is the primary hot topic on ASD. For some leading countries, intra country collaboration is dominant.

## Background

Autism spectrum disorders (ASD) represent a group of an early-onset neuro-developmental disorders that affects social interaction and communication abilities of affected people (Association AP 2013; Coleman and Gillberg 2011; Vinogradova 2015; Waterhouse 2013; Wiggins et al. 2015). ASD is an umbrella term that includes five different neuro-developmental disorders with varying degrees of severities, patterns and epidemiology. Such neuro-developmental disorders include: autism (autistic disorder), Asperger’s syndrome, pervasive developmental disorder not otherwise specified (PDD-NOS), childhood disintegrative disorder and Rett syndrome.

Autism Spectrum Disorders are universal affecting all racial, ethnic, and socioeconomic groups (Baio 2014). It is estimated that there are 52 million cases of ASDs worldwide (Baxter et al. 2015). The prevalence of ASD varies for each type of disorder and across different studies (Newschaffer et al. 2007). Data and statistics provided by Centers for Disease Control and Prevention (CDC) indicated that prevalence of ASD is increasing, however, a recent study argue against this finding and claimed that there is no clear evidence of a change in prevalence for autistic disorder or other ASDs between 1990 and 2010 (Baxter et al. 2015; Autism Spectrum Disorder, http://www.cdc.gov/ncbddd/autism/data.html).

ASD have negative economic consequences with real burden on families and governments. Furthermore, ASD have great emotional, social, and health impact on individuals with ASD and their families (Lavelle et al. 2014; Karst and Van Hecke 2012; Hayes and Watson 2013; Estes et al. 2013). A study indicated that autistic disorders accounted for more than 58 disability-adjusted life-years (DALYs) per 100,000 population while other ASDs accounted for 53 DALYs per 100,000 (Baxter et al. 2015). It has been reported that people with ASD had higher average medical expenditures than those without ASD (Shimabukuro et al. 2008). A study found that a child suffering from any kind of developmental disability imposes extra stress on his parents compared with children with no developmental disabilities (Karst and Van Hecke 2012). The stigmatization and discrimination led to the development of many social movements such as the autism rights movement (ARM) that encourages society to accept and deal with autism as a variation in functioning rather than a neurological or developmental disease to be cured by different therapeutic modalities (Jaarsma and Welin 2012).

A bibliometric profile of any medical topic allows researchers to acquire more knowledge about research trends, gives an insight into contribution of a particular country or institution to a given medical topic, and sheds more light into co-authorship and collaboration. Furthermore, bibliometric indicators such as citations and *h*-index can be used for university ranking, fund application and for scientific prestige among the scientific community. Many bibliometric studies have been published in various scientific disciplines and several ones were published about neurological disorders like multiple sclerosis, Parkinson’s disease and dementia (Li et al. 2008; Araujo et al. 2006; Aleixandre-Benavent et al. 2015; Gupta et al. 2013). The aim of this study was to analyze research output on ASD using a bibliographic analysis of articles indexed in Scopus.

## Methods

The data in this study were synthesized using Scopus database which has many important features that facilitate bibliometric analysis. Such features include citation analysis, country and author contribution as well as source titles and productivity per year. Scopus is produced by Elsevier and covers more than 20,000 journals that have 100 % Medline coverage. Scopus offers about 20 % more coverage than Web of Science, whereas Google Scholar offers results of inconsistent accuracy (Falagas et al. 2008). Since the data for this study was obtained from electronic sources that are publicly available and not pertaining to specific patients’ data or profile, IRB ethical approval for the study was not required.

The study period was set from January 01, 2005 to December 31, 2014. All subject areas in Scopus search engine (life sciences, social sciences, health and physical sciences) were chosen. All documents with the following title words: (TITLE (autism) OR TITLE (autistic) OR TITLE (“Asperger syndrome”) OR TITLE (“Asperger’s syndrome”) OR TITLE (“Rett syndrome”) OR TITLE (“childhood disintegrative disorder”) OR TITLE (“pervasive developmental disorder”) OR TITLE (“Heller’s syndrome”) OR TITLE (“disintegrative psychosis”) OR TITLE (“cerebroatrophic hyperammonemia”), plus all articles published in autism journals were retrieved and analyzed. The key words have been selected based on the broad definition of ASD and any alternative names available for any of the syndromes within the ASD definition. These key words are present in official web site related to ASD and other recent reviews about the subject (Autism, Autistic Spectrum Disorders (ASD) and Pervasive Developmental Disorders (PDD), http://www.med.umich.edu/yourchild/topics/autism.htm). To increase the accuracy of our search, all articles published in autism journals were included in the analysis. Furthermore, documents classified as errata, or books, or book chapter or un-defined type of documents or conference papers were excluded and therefore this study is restricted to documents that are considered journal articles. All documents obtained after refining the results were transferred to Statistical Package for Social Sciences software version 20 to present the bibliometric indicators. The validity of our method was assessed by assessing the first 100 top cited documents retrieved by the method mentioned above. All retrieved documents were in the ASD domain.

The main bibliometric indicators presented in this study included type and language of the published documents, country and institutional affiliation, source/journal title in which documents were published, most productive authors, most cited articles, and collaboration patterns (Sweileh et al. 2013, 2014; Zyoud et al. 2015a, b, c, d). The growth rate of publication per specific period was calculated as follows: (difference in number of articles published during that period/number of articles published at the start of the period) × 100. Many of the bibliometric indicators were presented in rank order. Research productivity was assessed by the quantity of publications while the total number of citations was used to identify the most influential articles in the field. The Scientific Journal Ranking (SJR) of journals was used as a measure of quality of journals and was obtained from SCImago Journal and Country Ranking website. The Hirsch index (*h*-index) was used to assess the quantity and quality of publications per country or per institution or per author. A country or an institution will have an h-index of x when it has published x papers, each of which has been cited at least x times to date (Hirsch 2005). The research productivity of different countries was normalized using population size and national gross domestic product (GDP) retrieved from the online databases of the World Bank (Countries and Economies, http://data.worldbank.org/country).

Bibliometric maps and visualization methods were made using VOSviewer software (van Eck and Waltman 2010). Using the VOSviewer and thresholds of minimally 10 fractionally counted articles for each term, density visualization maps were generated for most frequently encountered terms in title of retrieved articles. In these maps, most frequent terms had dense colored cluster. For co-authorship analysis, a minimum number of 500 authors were selected in VOSviewer program. Authors located within or close to a large cluster are believed to have higher number of co-authors suggestive of inter and intra country collaboration.

The whole data are available through Scopus and can retrieved using the search query in methodology. Furthermore, the whole data can be sent upon request to any interested researcher. The data can be sent either as a small file containing up to 2000 articles with full information pertaining to these articles. A second form is to send the whole data, 18,000 articles, with citations information only. The policy adopted by Scopus does not all the whole information of the whole data and allow up to 2000 articles to be exported. The exported file will be in an excel format.

## Results

### General data

During the specified time period, a total of 18,490 journal articles were retrieved. Of this number, 77.57 % were original articles while the remaining were review articles, letters, notes, editorials, short surveys and articles in press (Table 1). The primary language of retrieved articles was English (17,161; 92.81 %). Other languages like French, Italian, German, Spanish, Portuguese, Polish, Japanese, Chinese and Turkish were encountered. The total number of different languages encountered in the retrieved articles was 33. Table 2 shows the most commonly encountered languages.

**Table 1:** Types of documents of ASD publications (2005–2014)

Type of document	Frequency (%)
Article	14,343 (77.57)
Review	2214 (11.97)
Letter	600 (3.24)
Note	518 (2.8)
Editorial	396 (2.14)
Short survey	225 (1.22)
Article in press	194 (1.05)

**Table 2:** Top 10 languages of the retrieved articles on ASD (2005–2014)

Rank	Language	Total number of documents
1st	English	17,161 (92.81)
2nd	French	464 (2.51)
3rd	Spanish	201 (1.09)
4th	German	147 (0.8)
5th	Japanese	132 (0.71)
6th	Portuguese	117 (0.63)
7th	Chinese	82 (0.44)
8th	Polish	71 (0.38)
9th	Italian	65 (0.35)
10th	Turkish	45 (0.24)

### Most frequent terms

**Fig. 1:**
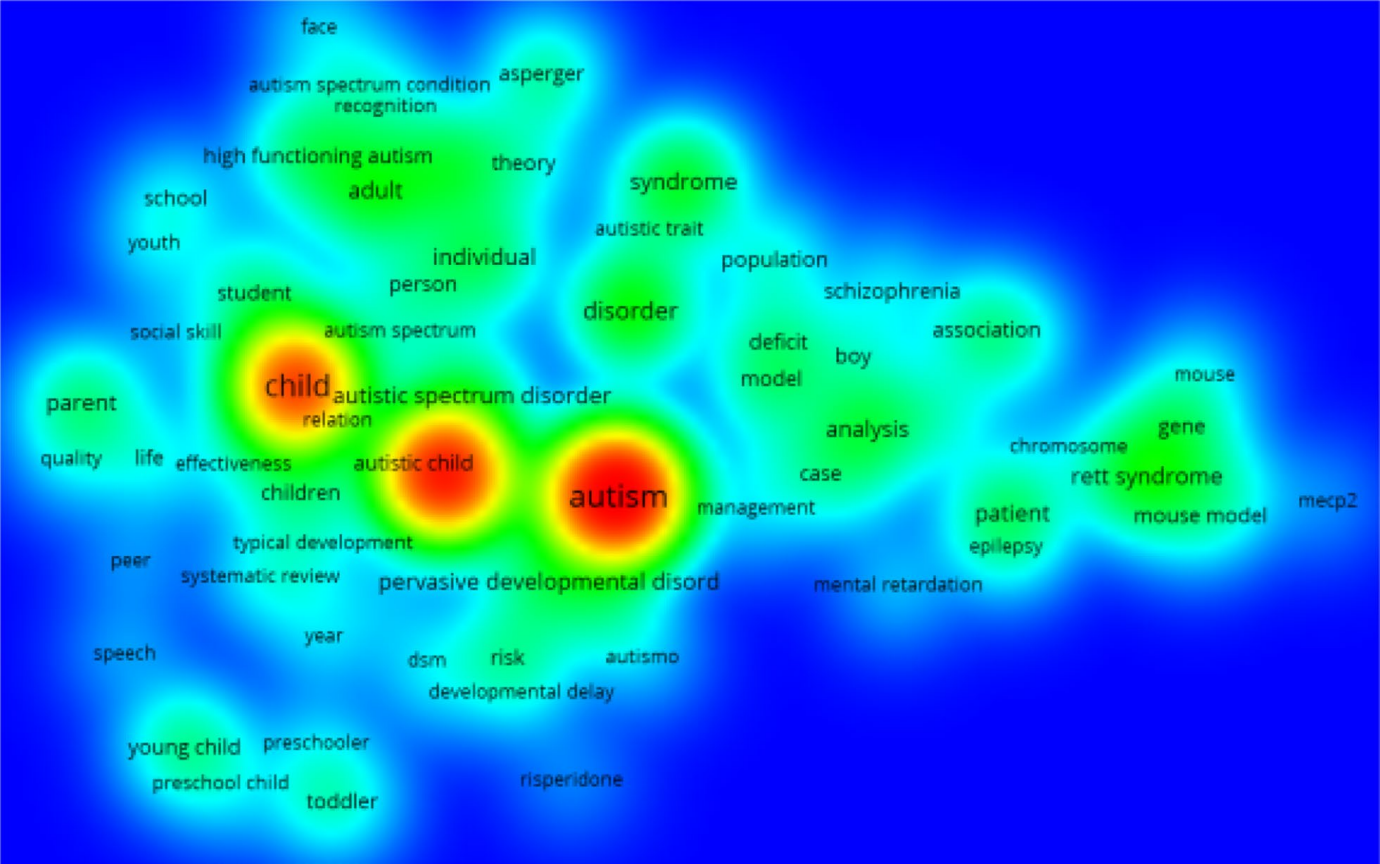
Density visualization map of frequently encountered title terms in retrieved articles on ASD (2005–2014)

In mapping terms frequency network, from the 27,427 terms, 142 terms met the threshold of 50 times as a minimum number of occurrences. Then 85 terms were selected as relevant terms based on calculated relevance score. Figure 1 shows the density visualization map of most frequently encountered terms in retrieved articles on ASD. For example, “autism” term was more commonly encountered than “pervasive developmental disorder” term in retrieved articles. Based on the map the following terms “autism = 6909 occurrences”, “autism spectrum disorder = 5182” and “child = 4395” had the highest frequency and were represented in three clusters in the density visualization map (Fig. 1).

### Publications with time

Approximately one third (6331; 34.24 %) of the retrieved articles were published in the first half of the study period while two thirds (12,159; 65.76 %) were published in the second half of the study period (2010–2014). A scatter plot showed a linear relationship between number of retrieved documents and time with an R^2^ = 0.987. Growth rate of publications from 2005 to 2014 was 229.15 %. Table 3 shows the number of retrieved articles per year as well as total number of citations, and number of citations per article. Articles published in 2005 had the highest average citations per article while the number of publications was highest in 2014. During the study period, the average number of different institutional affiliations per article was fluctuating within a range of 1.30–1.58 (Table 3).

**Table 3:** Number of published articles and citations on ASD during 2005–2014

Year	Total number of articles (%)	Total number of citations	Average number of citations per article	Average number of different institutional affiliations per article	Number of articles with zero citations (%)
2014	2992 (16.18)	11,755	3.93	1.49	844 (28.21)
2013	2753 (14.89)	22,018	8.00	1.47	512 (18.60)
2012	2424 (13.11)	33,982	14.02	1.30	373 (15.39)
2011	2147 (11.61)	38,461	17.91	1.40	283 (13.18)
2010	1846 (9.98)	42,384	22.96	1.39	225 (12.19)
2009	1614 (8.73)	44,593	27.62	1.48	193 (11.20)
2008	1509 (8.16)	47,548	31.50	1.47	185 (12.26)
2007	1246 (6.74)	49,429	39.67	1.58	147 (11.80)
2006	1050 (5.68)	45,550	43.38	1.43	119 (11.33)
2005	909 (4.92)	42,056	46.26	1.41	120 (13.20)

### Countries

A total of 102 countries contributed to the advancement of ASD research. Table 4 shows the top 10 productive countries in the field of ASD research along with their total population and GDP. The USA had the greatest share of publications in the field of ASD followed by UK, Canada and Australia. The USA was also the leading country in the annual number of publications from 2005 to 2014. The first three countries (USA, UK and Canada) shared almost two thirds (65.44 %) of the global research production in ASD field. Among the world regions, Northern America had the highest contribution (9671; 52.30 %) followed by Western Europe (6501; 35.16 %). The remaining world regions (Latin America, Asiatic region, Eastern Europe, Pacific Region, Middle East and Africa) contributed approximately 12 % to the world’s ASD publications. There was a significant correlation between number of retrieved articles in the top 10 productive countries and their total number of population (r = 0.903, p < 0.01) or GDP (r = 0.948, p < 0.01).

**Table 4:** Top 10 productive countries in number of ASD publications (2005–2014)

Rank	Country	Number of articles (%)	Number of articles per one million inhabitants (R)	Number of articles per one trillion of GDP (R)	Total citations	Number of citations per article	*h* index
1st	United States	8594 (46.48)	26.95 (6)	493.34 (6)	242,785	28.25	176
2nd	United Kingdom	2430 (13.14)	37.67 (3)	812.98 (2)	74,916	30.83	109
3rd	Canada	1077 (5.82)	30.30 (5)	603.36 (5)	33,618	31.21	85
4th	Australia	934 (5.05)	39.76 (2)	641.92 (4)	18,755	20.8	64
5th	Italy	782 (4.32)	12.75 (6)	365.25 (7)	19,126	24.46	61
6th	France	780 (4.22)	11.78 (8)	275.76 (8)	17,683	22.67	62
7th	Netherlands	632 (3.42)	37.51 (4)	718.75 (3)	17,868	22.27	60
8th	Japan	603 (3.26)	4.74 (10)	131.06 (10)	8759	14.52	46
9th	Germany	522 (2.82)	6.45 (9)	134.95 (9)	16,476	31.56	62
10th	Sweden	396 (2.14)	40.87 (1)	693.40 (1)	11,871	29.98	52

*R* rank

The total number of citations for the retrieved articles was 377,776 and the average number of citations per article was 20.43. Publications from USA had the highest share of citations (242,785) followed by those from UK (74,916) and Canada (33,618). However, publications from Germany had the highest number of citations per article (31.56) followed by those from Canada (31.21) and UK (30.83). When the h-index was used to assess the country impact of ASD publications, USA ranked first (176) followed by UK (109) and Canada (85) (Table 4).

### Inter and intra country collaboration

International (inter country) collaboration was also shown in Table 5. Articles from USA and Japan had the least percentage of inter-country collaboration calculated as percentage of multiple countries publication. For USA and Japan, more than 80 % of articles were published by domestic authors and presented as percentage of single country publication. On the other hand, more than half (56.56 %) of articles from Sweden, for example, had co-authors from other different countries. For the top 10 productive countries a total of 5055 articles had multiple country affiliation. Therefore, 30.18 % of published articles by the top 10 productive countries is a product of international collaboration and 69.82 % of the published articles were single country publications.

**Table 5:** List of top 10 productive countries on ASD research (2005–2014) with number of articles with multiple country or single country affiliation

Rank	Country	Number of articles (%) N = 18,490	Multiple country publication (%)	Single country publication (%)
1st	United States	8594 (46.48)	1675 (19.49)	6919 (80.51)
2nd	United Kingdom	2430 (13.14)	946 (38.93)	1484 (61.07)
3rd	Canada	1077 (5.82)	508 (47.17)	569 (52.83)
4th	Australia	934 (5.05)	368 (39.40)	566 (60.60)
5th	Italy	782 (4.32)	368 (47.06)	414 (52.94)
6th	France	780 (4.22)	286 (36.67)	494 (63.33)
7th	Netherlands	632 (3.42)	303 (47.94)	329 (52.07)
8th	Japan	603 (3.26)	117 (19.41)	486 (80.60)
9th	Germany	522 (2.82)	260 (49.81)	262 (50.19)
10th	Sweden	396 (2.14)	224 (56.56)	172 (43.43)
	Total	16,750 (90.59)	5055	11,695

### Authors

**Fig. 2:**
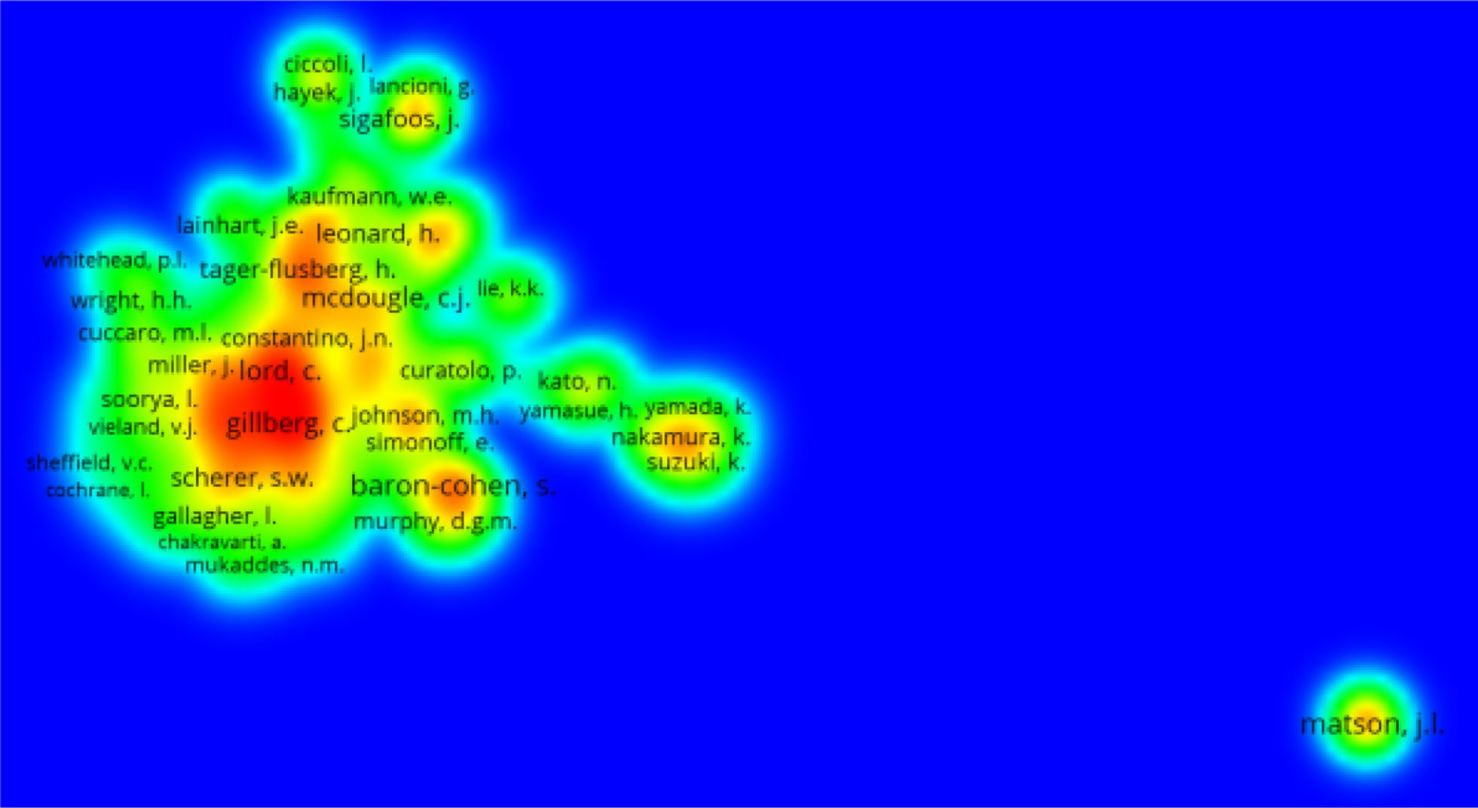
Density visualization map of co-authorship analysis for worldwide ASD research (2005–2014)

Professor Matson, J.L, from USA, ranked first in the number of publications with 228 articles (1.23 %) (Table 6). However, Baron-Cohen, S from UK ranked first in *h*-index with a value of 54. Of the most prolific authors, five were from USA, three were from the UK, one from Canada and one from Sweden. In this regard, it should be mentioned that a lot of debate and argument have been made regarding the role of self-citations in ranking top productive author in ASD. Density visualization of co-authorships using authors as unit of analysis showed that co-authorships were high and common among most prolific authors (Fig. 2). Co-authorships is suggestive of domestic and international collaboration. Authors who were remotely located from clusters have relatively fewer co-authorships and collaborations. It should be noted here that the list of top active authors is not based on the position of the author in the manuscript, whether first or last. Scopus counts the number of publications for each author regardless of his/her position in the manuscript. Scopus ranks authors based on their productivity regardless of their position in the manuscript. Therefore, the argument regarding first and last author analysis is not applicable here.

**Table 6:** Top 10 active authors publishing on ASD (2005–2014)

Rank	Author	Number of published articles	Total citation (R)	*h*-index (R)	Country
1st	Matson, J.L.	228	5376 (7)	39 (6)	USA
2nd	Baron-Cohen, S.	178	8273 (4)	54 (1)	UK
3rd	Gillberg, C.	139	8075 (5)	41 (5)	Sweden
4th	Minshew, N.J.	123	9477 (3)	46 (4)	USA
5th	Lord, C.	122	10,260 (2)	52 (2)	USA
6th	Charman, T.	119	5263 (8)	35 (8)	UK
7th	Dawson, G.	113	10,325 (1)	52 (2)	USA
8th	Zwaigenbaum, L.	98	7473 (6)	36 (7)	Canada
9th	McDougle, C.J.	93	5005 (9)	32 (8)	USA
10th	Happe, F.	90	4255 (10)	35 (10)	UK

*R* rank

### Frequently cited articles

The top 10 cited articles about ASD are presented in Table 7. The article which received the highest citation was “*Strong association of de novo copy number mutations with* *autism*” authored by Sebat et al. and published in 2007 in *Science* journal. The article received a total of 1427 citations up to the time of analysis of data (March 01, 2016). Six of the top 10 cited articles were in the field of genetic research.

**Table 7:** Top 10 cited articles on ASD (2005–2014) (Abrahams and Geschwind 2008; Baird et al. 2006; Dalton et al. 2005; Happe and Frith 2006; Marshall et al. 2008; Pinto et al. 2010; Sebat et al. 2007; Szatmari et al. 2007; Vargas et al. 2005; Weiss et al. 2008)

No.	Authors	Title	Year	Source title	Number of citations
1st	Sebat et al. (2007)	Strong association of de novo copy number mutations with autism	2007	*Science*	1427
2nd	Marshall et al. (2008)	Structural Variation of Chromosomes in Autism Spectrum Disorder	2008	*American Journal of Human Genetics*	908
3rd	Abrahams and Geschwind (2008)	Advances in autism genetics: On the threshold of a new neurobiology	2008	*Nature Reviews Genetics*	866
4th	Pinto et al. (2010)	Functional impact of global rare copy number variation in autism spectrum disorders	2010	*Nature*	862
5th	Weiss et al. (2008)	Association between microdeletion and microduplication at 16p11.2 and autism	2008	*New England Journal of Medicine*	848
6th	Szatmari et al. (2007)	Mapping autism risk loci using genetic linkage and chromosomal rearrangements	2007	*Nature Genetics*	842
7th	Baird et al. (2006)	Prevalence of disorders of the autism spectrum in a population cohort of children in South Thames: the Special Needs and Autism Project (SNAP)	2006	*Lancet*	826
8th	Happé and Frith (2006)	The weak coherence account: Detail-focused cognitive style in autism spectrum disorders	2006	*Journal of Autism and Developmental Disorders*	757
9th	Vargas et al. (2005)	Neuroglial activation and neuroinflammation in the brain of patients with autism	2005	*Annals of Neurology*	747
10th	Dalton et al. (2005)	Gaze fixation and the neural circuitry of face processing in autism	2005	*Nature Neuroscience*	680

**Fig. 3:**
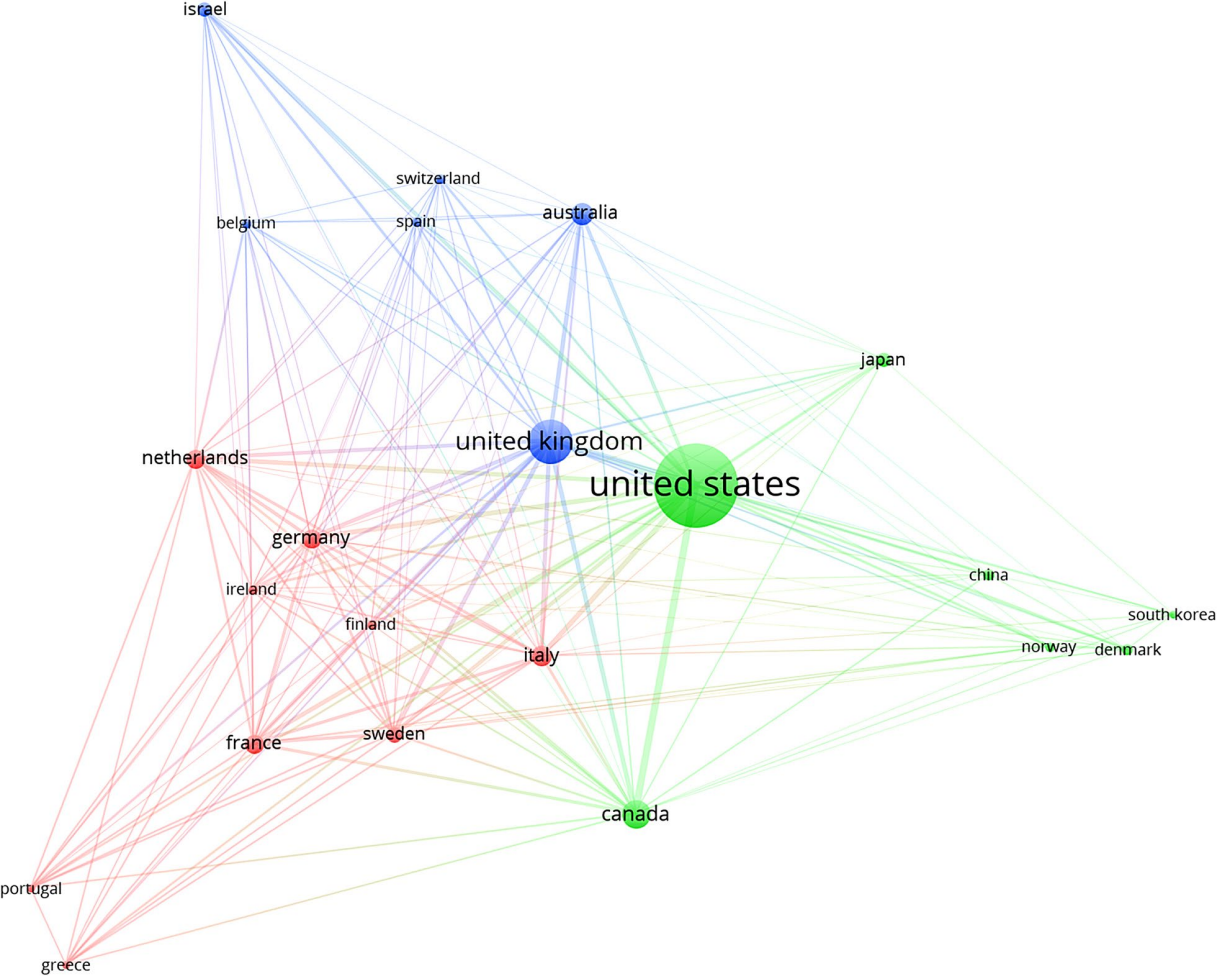
Network visualization map based on country co-authorship in most frequently cited articles on ASD (≥50 citations) during the period: 2005–2014

A total of 3001 (16.23 %) retrieved articles were not cited while 15,428 (83.44 %) were cited at least once. As expected, the zero citation was highest for articles published in 2014 compared to ones published in previous years. The number of articles which received at least 50 citations was 1951 articles (10.55 %). Network visualization of co-authorship in the most frequently cited articles for 22 selected countries based on a threshold of 10 is shown in Fig. 3. The network visualization is presented by circles and lines. The larger the circle, the greater the contribution of that country and the closer and thicker the lines are, the stronger and greater co-authorship between the connected countries (Fig. 3).

### Institutions

The first-ranking institution was King’s College London (UK) with 446 published articles (2.41 %) and an h-index of 68. Furthermore, King’s College London also ranked first in total citations. However, when institutions were ranked according to h-index, the University of California, Davis (USA) ranked first followed by King’s College London. American institutions occupied nine of the top 10 positions in the number of published articles on ASD from 2005 to 2014 (Table 8).

**Table 8:** Top 10 institutions publishing articles on ASD from 2005–2014

Rank (R)	Institution	Country	Number of published articles (%)	Total citations (R)	*h* index (R)
1st	King’s College London	UK	446 (2.41)	19,685 (1)	68 (2)
2nd	The University of North Carolina at Chapel Hill	USA	343 (1.86)	15,443 (4)	64 (3)
3rd	UC Davis	USA	327 (1.77)	16,877 (2)	72 (1)
4th	Yale Child Study Center	USA	290 (1.57)	13,859 (6)	62 (4)
5th	Vanderbilt University	USA	276 (1.49)	14,867 (3)	53 (7)
6th	Louisiana State University	USA	242 (1.31	5303 (9)	39 (10)
7th	University of California, Los Angeles	USA	238 (1.29)	12,605 (6)	56 (6)
8th	Harvard Medical School	USA	215 (1.16)	9471 (8)	52 (8)
9th	Massachusetts General Hospital	USA	214 (1.16)	10,209 (7)	50 (9)
10th	University of Washington Seattle	USA	213 (1.15)	15,425 (5)	61 (5)

*R* rank

### Journals

The retrieved articles were published in 1788 different journal names. A total of 4901 (26.59 %) articles were published in the top 10 productive journals (Table 9). The journal that has the largest share of publications was *Journal of Autism and Developmental Disorders* (n = 2050; 11.09 %). The journal that received the greatest number of citations was also the *Journal of Autism and Developmental Disorders*. However, the number of citations per article was greatest for *Journal of Child Psychology and Psychiatry and Allied Disciplines* (48.01) followed by *Journal of the American Academy of Child and Adolescent Psychiatry* (42.42). Table 9 also shows the SJR values for the top 10 productive journals. There was positive significant correlation between SJR values and the ratio of citations per article (r = 0.739, p = 0.015) for the top 10 productive journals. The highest SJR value in 2014 was that of *Journal of Child Psychology and Psychiatry and Allied Disciplines* which had an SJR value of 2.168 in 2005 and increased to 2.993 in 2014.

**Table 9:** Top 10 journals publishing in ASD (2005–2014)

Rank	Journal	Number of articles	Total number of citations	Number of citations per article	SJR 2014
1st	*Journal of Autism and Developmental Disorders*	2052	48,416	23.59	1.696
2nd	*Research in Autism Spectrum Disorders*	960	10,761	11.20	1.213
3rd	*Autism*	505	8911	17.64	1.255
4th	*Autism Research*	318	5980	18.80	1.994
5th	*Plos One*	266	4732	17.78	1.3
6th	*Research in Developmental Disabilities*	213	5775	27.11	0.986
7th	*Journal of Child Psychology and Psychiatry and Allied Disciplines*	165	7923	48.01	2.993
8th	*Molecular Autism*	151	2259	14.96	2.42
9th	*Journal of the American Academy of Child and Adolescent Psychiatry*	136	5770	42.42	2.813
10th	*Focus on Autism and Other Developmental Disabilities*	135	1244	9.21	0.91

*SJR* SCImago Journal rank indicator

## Discussion

In the current study, we aimed to investigate the scientific research on ASD using bibliometric indicators for articles published during 2005–2014. Scopus was used to achieve the objective of this study. Scopus is considered a larger database than Web of Science and has several advantages over Medline like availability of citation analysis and many other bibliometric parameters (Falagas et al. 2008).

The worldwide growth in ASD research should be considered within the following points: there is an overall increase in various medical and biomedical research topics in the last few decades facilitated by increased number of specialized journals, advancement of molecular genetics technology, and increased governmental and non-governmental funding for unsolved mysteries of several diseases. Several publications have indicated that there is a prominent increase in neurology and psychiatry research topics like Multiple Sclerosis, Epilepsy, Schizophrenia, Anxiety, Dementia and others (Araujo et al. 2006; Aleixandre-Benavent et al. 2015; Gupta et al. 2013; Morandi et al. 2015). It should be emphasized that part of the growth of ASD research is due to the social health consequences of the ASD. Many international societies have been established to support research and to support families with ASD children. Examples of such international associations include the International Society for Autism Research and Autism Network International. Finally, the presence of articles with non-English language is also an indicator of worldwide growing interest in the scientific and social aspects of ASD.

The global increase in number of annual publications on ASD was not accompanied by an increase in inter-institutional collaboration and the number of different institutional affiliation per article with time remained approximately constant. Again, this suggests that the majority of research publications in ASD research is led by research groups affiliated with one institution. It seems that well established research centers in autism tend to interact domestically with authors within the same country because international collaboration might be of limited benefit for such well-established research centers. University College London in UK had the greatest share of publications, total citations and *h*-index value. Furthermore, UK had the greatest share when the number of publications was normalized with GDP. However, USA with so many active institutions ranked first with a total of 8594 articles and an *h*-index of 176. Of the top 10 countries, none of the countries were from Latin America or East Europe or Africa, or Middle East. It should be noted here that although the data was stratified by population size and GDP, still, the interpretation of data pertaining to country list needs to take into consideration the guidelines adopted for each country in the diagnosis of ASD and the governmental and non-governmental funding available for research centers and researchers to carry out research on ASD.

Publications from authors affiliated with USA or Japan, or UK or Canada showed dominant domestic collaboration. On the hand, publications from authors affiliated with Italy, France, Australia, Netherland, and Sweden showed dominant international country collaboration. Publications with author from Germany were split in equal between domestic and international country collaboration. International collaboration in ASD research and publication should be encouraged and emphasized given the fact that ASD affects all races and cultures all over the world. Collaboration in social, psychological and therapeutic research could be established between developed and developing countries. A bibliometric study on sleep apnea showed that the probability of citation increased by 1.23 times for each additional author, and by 2.23 times for each additional country represented; independent of time since publication, journal, or the country of the author (Huamani et al. 2015).

The top 10 cited articles revealed that more than half of the hot articles were focused on molecular genetics and cytogenetics of ASD. The other ones were related to epidemiology and pathophysiological characteristics of ASD. The genetics of autism and other ASD remain unsolved and extensive research is being carried out to understand the molecular aspects of these disorders and that is why most of genetic articles about ASD received high number of citations. No wonder that such articles were published in highly prestigious journals that are not in the specific field of autism. As expected, autism journals dominated the top 10 list of journals publishing on ASD during the study period. However, psychology journals like *Journal of Child Psychology and Psychiatry and Allied Disciplines* which published on psychological aspects of ASD has reached international recognition and audience with an impact factor of approximately 6.5 and an average citation per doc of 48 per article. Of a considerable interest is *PLOS ONE* journal which ranked 5th in publications about ASD. *PLOS ONE* is a multidisciplinary, open access journal, with broad scope which explains the large quantity of ASD articles in this journal. An important final note regarding highly cited articles is that it is possible that number of geneticists is greater than number of psychologists and family therapists in most countries which made genetic research occupies most of highly cited articles.

Our study showed that the number of uncited articles represents 16.23 % which is relatively higher than that reported in a similar bibliometric study about Multiple Sclerosis (Aleixandre-Benavent et al. 2015). However, the number of citations of any article varies from time to time and from one journal to another. Therefore, comparison of uncited articles from one subject to another might not be of great benefit. Regarding the most cited articles, it was about number of mutations and it association with autism. The article was published in 2007 in *Science* journals. The second most cited article was also about chromosomal variations and autism and was published in 2008 in *American Journal of Human Genetics*. It is noteworthy that the journals in which the first seven most cited articles were non-neurology, non-psychiatry journals, but ones with extremely high impact factor like *Science* and *Nature* journals.

Our study has a few limitations related to search strategy and methodology (Sweileh et al. 2013, 2014; Zyoud et al. 2015a, b, c, d). For example, our study did not include articles published in non-Scopus database. However, Scopus remain a reliable source for bibliometric studies in general. Another limitation in our study is the keywords strategy. False positive and false negative results could be obtained regardless of how accurate the search stagey was. With a total of more than 18,000 articles, we believed that false positive or negative results will be very marginal and could hardly affect the accuracy of the results. Furthermore, the use of the keywords in title search instead of title/abstract/keywords would minimize false positive and negative articles and keep non-relevant articles in the minimum tolerable number. There are several journals specialized in autism and we thought that it is logical to include all articles published in these journals regardless of keywords used in the title of articles published in these journals. One might argue against such strategy, but we thought it will be unfair not to include all articles published in these special journals. Finally, the indicators used to assess quality of publications need to be considered carefully. There is no direct measure for quality of publications. However indirect measures like citations, *h*-index, and IF have been suggested as indirect indicators for quality. The *h*-index is based on the number of citations received by a certain author, institution, journal, or country. The number of citations is complicated by self-citations which created a lot of bias in assessing quality and impact of publications by authors (Nightingale and Marshall 2013; Gaster and Gaster 2012). Therefore, names of authors listed in the manuscript might not really reflect a true rank and a deeper analysis regarding self-citations of top ranking authors need to be examined. Such bias in self-citations have been the focus of debate regarding certain authors in certain medical field including autistic disorders (Tolisano et al. 2016). A study has shown that the h-index is not a valid parameter to differentiate between assistant professor and associate professor ranks or between MD’s and PhD’s (Doja et al. 2014). A recent study has discussed the merits and demerits of various parameters used to assess quality of publications and suggested “Original Research Performance Index (ORPI)” for evaluation of an author’s original research which can minimize bias arising because of self-citations, gift authorship, inactive phase of research, and length of non-productive period in research (Saxena et al. 2013). Some authors suggested the use of three factors; IF, Eigenfactor Score (ES), and SCImago Journal rank indicator (SJR) when judging quality of the certain medical journals to overcome the several shortcomings of IF (Ramin and Sarraf Shirazi 2012). A very recent editorial in *Nature* described the use of IF as crude and misleading and that the best assessment of publications is not the journal metrics, rather, reading the paper itself and forming the opinion is the best judgment (Nature Publishing Group 2016).

## Conclusion

To the best of our knowledge, this is the first bibliometric study on ASD. The results of our study showed the following characteristics regarding ASD research and publications: there is a growing interest in this topic as seen by the linear increase in the number of publications with time; there is a dominant leadership for Northern Americas and Western Europe in ASD publications; there is a wide variety of journal names in which ASD articles are published; there is a great focus on molecular genetics in ASD research as demonstrated by the title of hot articles in the field; and there is a wide variation in inter-country collaboration in ASD research among the top leadership countries like USA and UK and there is a common trend toward domestic different countries.

## Abbreviations

ASD: autism spectrum disorders
GDP: gross domestic product
*h*-index: the Hirsch index
IF: impact factor
IRB: Institutional Review Board
SCR: Standard Competition Ranking
USA: Unite States of America
UK: United Kingdom
